# Takeaway Fast-Food Consumption and Elevated Diastolic Blood Pressure in US Young Adults: An Exploratory Mediation Analysis of Metabolic and Inflammatory Indicators

**DOI:** 10.3390/healthcare14142059

**Published:** 2026-07-09

**Authors:** Gaozhao Chu, Jun Chen, Lihong Wang

**Affiliations:** 1 Second Clinical Medical College, Zhejiang Chinese Medical University, Hangzhou 310053, China; 202411121611017@zcmu.edu.cn; 2 Department of Cardiology, Zhejiang Provincial People’s Hospital, People’s Hospital of Hangzhou Medical College, 158 Shangtang Road, Hangzhou 310014, China; 21818354@zju.edu.cn

**Keywords:** takeaway fast-food consumption, diastolic blood pressure, mediation analysis, insulin resistance, dietary inflammatory index

## Abstract

**Background:** Global takeaway fast-food consumption (TFC) has surged. This study aimed to investigate the association between TFC frequency and diastolic blood pressure (DBP) and to explore the potential mediating roles of metabolic and inflammatory markers. **Methods and Results:** In this cross-sectional study of 13,062 adults from the National Health and Nutrition Examination Survey (NHANES), we utilized weighted regression and restricted cubic splines (RCS) to evaluate the association between TFC frequency and DBP. We performed exploratory mediation analyses to explore the roles of body mass index (BMI), visceral adiposity index (VAI), cardiometabolic index (CMI), dietary inflammatory index (DII), and insulin resistance (HOMA-IR). In the overall cohort, the main association between TFC frequency and DBP did not reach statistical significance in fully adjusted models. However, restricted cubic spline analysis revealed a linear dose–response trajectory without an evident threshold effect. In exploratory subgroup analyses, a potentially pronounced positive association was noted among females, although the formal interaction test was not statistically significant. Mediation models indicated that the examined metabolic and inflammatory markers did not significantly mediate this relationship, suggesting that the association with diastolic blood pressure may not operate through these specific pathways. **Conclusions:** While the overall main categorical association did not reach statistical significance, TFC demonstrated a potential continuous trend with DBP, with potential associations observed among females. Notably, this relationship was not mediated by established metabolic or inflammatory indices, suggesting that the association between TFC and blood pressure elevation may exist independent of overt structural obesity. These findings highlight the potential value of incorporating takeaway fast-food consumption into routine dietary screening for early cardiovascular risk assessment in young adults.

## 1. Introduction

Hypertension remains the leading preventable cause of premature death and cardiovascular disease (CVD) worldwide, with its global prevalence having doubled over the past three decades [1]. Historically regarded as an age-related disorder, hypertension is now showing a concerning upward trend among younger and middle-aged demographics, according to contemporary epidemiological data [2]. In these younger demographics (typically aged <50 years), the hemodynamic profile differs distinctly from that of the elderly: isolated diastolic hypertension (IDH) is the predominant phenotype, and elevated diastolic blood pressure (DBP) is increasingly recognized as an important and independent contributor to subsequent cardiovascular events [3]. Despite its prognostic significance, DBP is frequently overshadowed by the clinical emphasis on systolic hypertension. Emerging large-scale cohort studies have consistently affirmed that higher DBP independently elevates the susceptibility to myocardial infarction and stroke, necessitating urgent attention to diastolic blood pressure control for early cardiovascular prevention [4].

Coinciding with rapid urbanization and the expansion of convenient commercial dining options, takeaway fast-food consumption (TFC)—which in this study is predominantly characterized by meals sourced from fast-food restaurants and pizza places—has evolved from an occasional treat to a routine dietary behavior globally [5]. While these ready-to-eat meals offer convenience, the nutritional quality of these takeaway meals remains a major public health concern. Comprehensive nutritional profiling indicates that such commercial fast-food and takeaway options are typically energy-dense and nutrient-poor, characterized by excessive amounts of total fat, saturated fatty acids, and refined carbohydrates compared to home-cooked meals [6,7]. As a result, habitual TFC is robustly associated with a spectrum of detrimental health consequences, spanning obesity, metabolic syndrome, and greater overall mortality [8].

Despite the robust evidence linking takeaway fast-food to weight gain, the specific relationship between TFC and blood pressure profiles—particularly diastolic blood pressure—remains under-investigated. While some studies have established the link between processed food intake—a major source of dietary sodium—and elevated blood pressure [9], most prior research has failed to differentiate between systolic and diastolic components. Crucially, a significant knowledge gap exists regarding the underlying potential mediators. The prevailing paradigm attributes diet-induced hypertension largely to the mediation of excess adiposity and metabolic dysfunction, such as insulin resistance and systemic inflammation [10,11]. However, this “obesity-centric” view may obscure independent pathogenic pathways. While the long-term obesogenic impact of fast food is well-established, it remains largely unknown whether high-frequency consumption exerts an early, independent hemodynamic burden prior to the onset of clinically evident metabolic derangement. Given the substantial sodium load and potential additives in takeaway meals, we hypothesize that TFC might contribute to blood pressure elevation through alternative, unmediated routes. Potential explanations for this include sodium-induced volume expansion, endothelial dysfunction, or sympathetic activation, which could theoretically operate independently of body mass index (BMI) [12,13]. While these specific mechanisms remain speculative at this stage due to the observational nature of epidemiological data, untangling these pathways is essential. To date, no large-scale study has utilized mediation analysis to quantify the extent to which the TFC-DBP association is mediated by metabolic factors versus unmediated associations, limiting the development of precise preventative strategies.

To bridge this literature gap, our research analyzed information from the National Health and Nutrition Examination Survey (NHANES), an extensive, population-based assessment reflecting the demographic makeup of the United States [14]. We specifically focused on adults aged under 50 years, a critical demographic window where elevated blood pressure—including isolated diastolic hypertension—is increasingly recognized as a potent predictor of premature cardiovascular events, yet it often remains undertreated [15]. This specific age restriction was predicated on both behavioral and pathophysiological considerations. Epidemiologically, younger and middle-aged adults represent the primary demographic consuming takeaway meals, largely driven by fast-paced lifestyles and the inherent convenience of fast food [16]. Pathophysiologically, DBP trajectories exhibit distinct age-dependent patterns: DBP generally rises linearly until approximately the fifth decade of life, after which it tends to plateau or decline due to progressive age-related large artery stiffening [17]. Furthermore, isolated diastolic hypertension is the predominant hypertensive phenotype in individuals under 50, whereas isolated systolic hypertension dominates in older populations [18]. Therefore, focusing on individuals under 50 years of age minimizes the confounding effects of senescent vascular aging on DBP and allows for a clearer elucidation of the relationship between contemporary dietary habits and early-stage blood pressure elevation. Beyond evaluating the dose–response relationship, we employed a comprehensive mediation analysis framework to disentangle the pathways linking TFC to DBP. We systematically quantified the potential mediating roles of traditional obesity metrics (BMI) [19], novel visceral adiposity indicators (VAI, CMI) [20,21], systemic inflammation (DII) [22], and insulin resistance (HOMA-IR) [23]. Our primary objective was to determine whether the observed relationship between TFC and blood pressure is dependent on metabolic dysfunction or if it represents an unmediated, independent link, thereby providing precise evidence for early dietary intervention.

## 2. Methods

### 2.1. Study Design and Population

Data for the current analysis were derived from the NHANES, an ongoing cross-sectional surveillance system established to monitor the clinical and dietary profiles of the non-institutionalized American public. NHANES employs a complex, multistage, stratified probability sampling design to ensure national representativeness, with data released in two-year cycles [24]. Following the acquisition of informed consent under the oversight of the National Center for Health Statistics (NCHS) Research Ethics Review Board, enrollees completed household questionnaires and subsequently received physiological and biochemical evaluations at Mobile Examination Centers (MECs). For the current analysis, we aggregated five survey cycles spanning from 2009 to 2018. After excluding individuals with missing data on takeaway fast-food consumption or blood pressure measurements, the final analytical sample consisted of 13,062 adults.

### 2.2. Sociodemographic Characteristics

**Figure 1:**
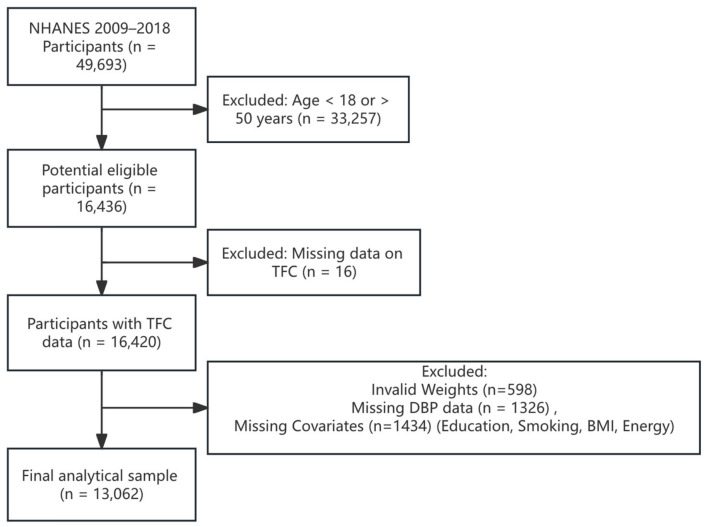
Flowchart of study participant selection from NHANES 2009–2018.

The NHANES 2009–2018 dataset initially included 49,693 participants. For this analysis, a total of 13,062 individuals were included. The age restriction to adults under 50 years was pre-specified to minimize the confounding impact of senescent vascular aging on large arteries, which typically alters DBP trajectories after the fifth decade of life, thereby allowing for a clearer elucidation of dietary factors on early-stage isolated diastolic hemodynamic profiles. Initially, 33,257 participants aged <18 years or >50 years were excluded. Subsequently, another 3374 participants were excluded due to missing data on key variables, including TFC (*n* = 16), sample weights (*n* = 598), DBP (*n* = 1326), and covariates (*n* = 1434) (detailed screening process shown in Figure 1). The NHANES study protocol was originally approved by the NCHS Research Ethics Review Board, and documented informed consent was obtained from all participants prior to their examination.

### 2.3. Takeaway Food Consumption (TFC)

Because terminology such as “takeaway fast-food,” “fast food,” and “online food delivery” can vary conceptually and internationally, we explicitly established an operational definition for this study. Here, TFC is operationally defined and measured as the consumption of meals from fast-food restaurants or pizza places. Participants were asked, “During the past 7 days, how many times did you eat food from fast-food restaurants or pizza places?” While contemporary takeaway behavior encompasses a diverse range of online delivery options, fast-food and pizza establishments represent the most foundational, prevalent, and highly representative sources of commercial takeaway meals during the NHANES survey cycles analyzed. The reported frequencies were converted into a continuous variable (times/week). To facilitate subsequent evaluations, the study population was stratified into four distinct groups according to their weekly intake: never/rare (0–1 times/week), low (2–3 times/week), moderate (4–5 times/week), and high (≥6 times/week).

### 2.4. Assessment of Blood Pressure and Hypertension

Within NHANES, blood pressure measurements were primarily obtained at MECs. Each eligible participant underwent three to four seated systolic and diastolic blood pressure measurements administered by trained examiners using standardized protocols.

During MEC examinations, mercury sphygmomanometers were used in strict accordance with the technical recommendations of the American Heart Association [25]. Beginning in 2017, blood pressure was measured using the automated Omron HEM-907XL device (Omron Corporation, Kyoto, Japan), which records three consecutive readings at 60 s intervals [26]. This device has been validated according to standards established by the Association for the Advancement of Medical Instrumentation and the European Society of Hypertension and is approved for use in individuals aged 13 years and older [27]. When multiple readings were available, the average value was used for analysis. In accordance with the European Society of Cardiology guidelines, participants were classified as hypertensive if they exhibited an average systolic/diastolic blood pressure of ≥140/≥90 mmHg, were actively taking blood pressure-lowering medications, or reported a prior clinical diagnosis [27].

### 2.5. Assessment of Potential Mediators

To systematically evaluate the potential biological mechanisms linking takeaway fast-food consumption to blood pressure, we analyzed five specific metabolic and inflammatory indicators. BMI was established by dividing the individual’s weight (kg) by their squared height (m^2^). Visceral adiposity was evaluated using the VAI, a sex-specific indicator incorporating waist circumference (WC), BMI, triglycerides (TG), and high-density lipoprotein cholesterol (HDL-C), calculated based on the formula established by Amato et al. [20]. The CMI, an emerging metric for cardiovascular risk stratification, was computed by multiplying the waist-to-height ratio (WHtR) by the TG/HDL-C ratio [22]. Insulin resistance was assessed using the HOMA-IR, calculated as (fasting insulin [μU/mL] × fasting glucose [mmol/L])/22.5 [23]. Furthermore, to evaluate the overall inflammatory capacity of the participants’ diets, we derived the DII from 24-h dietary recall information. This derivation involved normalizing the available nutritional components against an established global reference dataset [22].

### 2.6. Assessment of Covariates

The inclusion of potential confounders was strictly guided by their mechanistic relevance and well-documented links to elevated blood pressure. Sociodemographic variables included age, sex, race (categorized as Mexican American, Other Hispanic, Non-Hispanic White, Non-Hispanic Black, and Other Race including Multi-Racial), educational attainment (Less than 9th grade, 9–11th grade, High school graduate/GED, Some college or AA degree, College graduate or above), and family income-to-poverty ratio (PIR). Lifestyle factors consisted of smoking status and physical activity levels. Anthropometric and clinical measurements included weight (kg), height (cm), waist circumference (cm), and pulse (times/min), which were measured by trained health technicians following the standardized protocols described in the NHANES Plan and Operations [24]. Biochemical profiles were analyzed from fasting blood samples collected at the MEC, including fasting glucose (FG) (mmol/L), serum insulin (pmol/L), glycosylated hemoglobin (HbA1c%), total cholesterol (TC) (mmol/L), HDL-C (mmol/L), TG (mmol/L), low-density lipoprotein cholesterol (LDL-C) (mmol/L), and serum creatinine (umol/L). All laboratory analyses were conducted according to the rigorous methods detailed in the NHANES Laboratory Procedures Manuals [28].

## 3. Statistical Analysis

All statistical analyses were performed using R software (version 4.3.3). Given the complex, multistage probability sampling design of NHANES, sample weights were appropriately applied in all analyses. Participants with missing data on essential variables were excluded using listwise deletion to construct the final analytical cohort. Specifically, the full-sample MEC weights were applied for demographic, socioeconomic, and routine clinical variables. Conversely, for metabolism-related indicators exclusively measured in the randomly assigned morning fasting subsample (including fasting glucose, serum insulin, triglycerides, LDL-C, VAI, CMI, and HOMA-IR), the designated fasting subsample weights were strictly applied to produce nationally representative estimates.

Continuous metrics are reported as weighted average values, while categorical data are represented by weighted percentages; all estimates are provided with 95% confidence intervals (CIs). Weighted linear regression was used to examine the distributions of continuous baseline features across TFC tiers, while the Rao–Scott adjusted Chi-square test was applied to analyze categorical variables.

To assess the relationship between TFC and blood pressure metrics (expressed in clinically interpretable units of mmHg for continuous variables), we performed survey-weighted linear and logistic regression analyses to derive coefficients and odds ratios (ORs), respectively. Potential confounders were managed through three incrementally adjusted multivariable models: a crude model (Model 1); an initial adjustment for demographic factors (age, sex, and race; Model 2); and a fully adjusted model further incorporating socioeconomic and lifestyle variables (education, PIR, smoking, physical activity, and BMI; Model 3). BMI was included in Model 3 to initially evaluate the independent association between TFC and blood pressure, distinct from its subsequent evaluation as a specific mediator in the causal mediation framework. Linear trends were evaluated by assigning the median of each TFC quartile to a continuous term in the regression models.

To evaluate the potential presence of non-linearity in the association between continuous TFC and DBP, we implemented a survey-weighted Restricted cubic splines (RCS) approach. The spline function was configured with three knots—positioned at the 10th, 50th, and 90th percentiles—to accommodate any curvilinear patterns.

Subgroup analyses and interaction tests were performed to examine whether the associations varied by age (<45 vs. ≥45 years), sex, BMI status, smoking status, and diabetes status. The significance of interactions was evaluated by including a cross-product term of the stratification factor and TFC in the fully adjusted models.

Furthermore, statistical mediation analyses were conducted to quantify the extent to which metabolic and inflammatory markers (including BMI, VAI, CMI, DII, and HOMA-IR) mediated the association between TFC and DBP. These specific indices were purposively selected to comprehensively capture distinct pathophysiological dimensions: generalized adiposity (BMI), visceral lipid accumulation and function (VAI, CMI), dietary-induced systemic inflammation (DII), and insulin resistance (HOMA-IR). The total effect (TE), average direct effect (ADE), and average causal mediation effect (ACME) were estimated. Because this study utilizes a cross-sectional design, temporal ordering cannot be established; therefore, these analyses strictly represent statistical mediation patterns rather than definitive causal biological pathways. The statistical significance of the mediation effects was determined using a bootstrapping method with 1000 resamples. A two-sided *p*-value < 0.05 was considered statistically significant.

## 4. Results

### 4.1. Description of Baseline Characteristics

A total of 13,062 participants were included in the final analysis. Participants were categorized into quartiles based on their weekly TFC frequency: Q1 (0–1 times/week, *n* = 5247), Q2 (2–3 times/week, *n* = 4018), Q3 (4–5 times/week, *n* = 2269), and Q4 (≥6 times/week, *n* = 1528).

Table 1 summarizes the baseline demographic and clinical profiles of the analytical cohort, stratified by TFC quartiles. Compared to participants in the lowest TFC quartile (Q1), those with higher TFC frequency (Q4) were significantly younger (mean age: 34.01 vs. 35.44 years, *p* = 0.001) and more likely to be male (59.4% vs. 48.8%, *p* < 0.001). Socioeconomically, high-frequency consumers had higher educational attainment (college graduate or above: 39.0% vs. 27.3%, *p* < 0.001) and a higher family poverty income ratio (PIR: 3.15 vs. 2.60, *p* < 0.001).

Regarding anthropometric and biochemical profiles, participants in the highest quartile (Q4) had significantly higher weight (*p* = 0.019), height (*p* < 0.001), and serum creatinine levels (*p* < 0.001).

Notably, despite the higher frequency of takeaway consumption, there were no statistically significant differences in baseline systolic blood pressure (*p* = 0.632), diastolic blood pressure (*p* = 0.192), or the prevalence of hypertension (*p* = 0.457) across the TFC quartiles. Similarly, no significant differences were observed for smoking status, physical activity, waist circumference, fasting glucose, HbA1c, HDL-C, or other lipid profiles (all *p* > 0.05). Such crude results suggest that any potential association between TFC and blood pressure variations might be confounded by the demographic advantages (e.g., younger age and higher socioeconomic status) of high-frequency users, thus highlighting the necessity for rigorous multivariable adjustment in subsequent analyses.

### 4.2. Associations Between TFC Frequency and Blood Pressure Outcomes

The relationship between TFC frequency and the odds of hypertension, as well as its impact on linear blood pressure metrics, is detailed in Table 2, Table 3 and Table 4.

In the survey-weighted logistic regression analyses, no significant association was observed between TFC frequency and the prevalence of hypertension. As shown in Table 2, compared with participants in the lowest quartile (Q1), the multivariable-adjusted ORs (95% CIs) for those in Q4 were 1.03 (0.84, 1.27) in the fully adjusted Model 3 (*p* = 0.744). Furthermore, the assessment of linear trends yielded no evidence of statistical significance across the categorical tiers $(P_{\mathrm{trend}}\;=\;0.666)$. Regarding continuous blood pressure traits, linear regression analyses revealed no significant association between TFC quartiles and Systolic Blood Pressure (SBP) (β = 0.16, 95% CI: −0.64 to 0.96 for Q4 vs. Q1; *p* = 0.687; Table 4).

For DBP, although the overall main effect and linear trend did not reach statistical significance in the fully adjusted model (*p* for trend = 0.327, Table 3), the regression coefficients demonstrated a numerically progressive increase from Q1 to Q4. Specifically, participants with the highest TFC frequency exhibited a marginally higher DBP level (β = 0.53, 95% CI: −0.29 to 1.36 for Q4 vs. Q1) compared to the lowest frequency group. This positive shift in β coefficients following multivariable adjustment suggests that the crude baseline similarities in DBP were likely confounded by the demographic advantages of high-frequency consumers (e.g., younger age and higher socioeconomic status), which initially masked a potential positive association. Nevertheless, the absence of a statistically significant main effect across the general young adult population highlights the necessity of performing subsequent subgroup analyses to identify potentially vulnerable subpopulations.

### 4.3. Subgroup Analyses

To further explore potential effect modifiers, we performed exploratory subgroup analyses stratified by age, sex, BMI, smoking status, and diabetes status.

**Figure 2:**
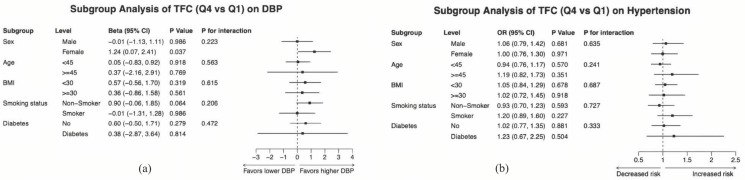
Subgroup analyses of takeaway fast-food consumption (Q4: ≥6 vs. Q1: 0–1 times/week) on blood pressure outcomes. (**a**) Survey-weighted β coefficients and 95% CIs for diastolic blood pressure (DBP). (**b**) Multivariable-adjusted odds ratios (ORs) and 95% CIs for the prevalence of hypertension. All models were adjusted for age, sex, race/ethnicity, education, poverty income ratio (PIR), smoking status, physical activity, and BMI (stratification variables were excluded from corresponding models). *p* for interaction indicates the significance of subgroup differences. Abbreviations: TFC, takeaway fast-food consumption; DBP, diastolic blood pressure; CI, confidence interval; OR, odds ratio; BMI, body mass index.

Figure 2a illustrates the multivariable-adjusted subgroup analyses for DBP. In exploratory subgroup analyses, a potentially pronounced positive association between high TFC frequency (Q4) and DBP was noted among females (β = 1.24, 95%CI: 0.07 to 2.41); however, these stratum-specific findings should be interpreted cautiously, as the formal test for interaction by sex was not statistically significant (*p* for interaction = 0.223). Additionally, no statistically significant associations were observed when stratified by smoking status (e.g., non-smokers: β = 0.90, *p* = 0.064), diabetes status, or other baseline characteristics. Given the lack of statistically significant effect modification (all *p* for interaction > 0.05), these observations should be interpreted strictly as exploratory findings rather than confirmatory evidence of subgroup-specific susceptibility.

Figure 2b presents the subgroup analyses for the prevalence of clinical hypertension. Consistent with our findings in the main cohort, the associations between high TFC and hypertension remained uniformly non-significant across all stratified subgroups (all *p* > 0.05).

### 4.4. Restricted Cubic Spline Analysis

**Figure 3:**
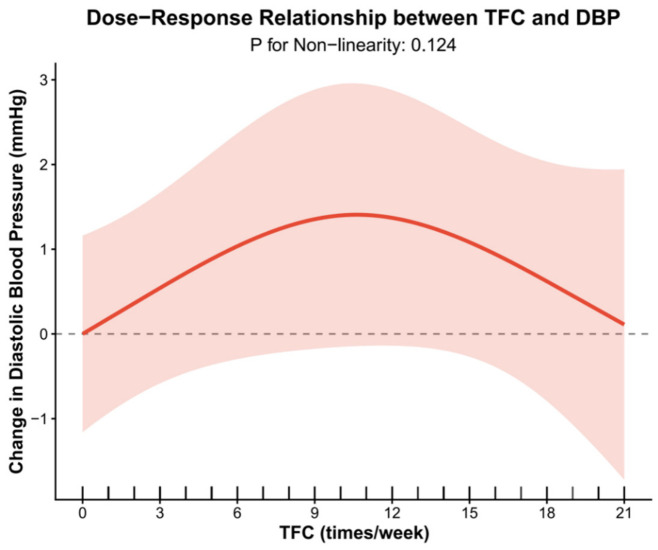
Dose–response relationship between takeaway fast-food consumption frequency and diastolic blood pressure. Note: 95% confidence interval (CI). The model was adjusted for age, sex, race, education, poverty income ratio, smoking status, physical activity, and body mass index. The dashed line represents the reference level. $P_{\mathrm{non}-\mathrm{linearity}}\;=\;0.093$, indicating a generally linear relationship. Abbreviations: TFC, takeaway fast-food consumption; DBP, diastolic blood pressure.

To further explore the shape of the association between takeaway fast-food consumption and blood pressure levels, we performed a survey-weighted RCS analysis utilizing the fully adjusted multivariable model (Model 3). Figure 3 illustrates the dose–response relationship between the continuous frequency of TFC and DBP levels. Following rigorous adjustment for demographic, socioeconomic (including Poverty Income Ratio), and lifestyle covariates, the test for non-linearity was non-significant (*p* for non-linearity = 0.124). Importantly, this lack of significance primarily indicates no clear evidence of non-linearity, rather than definitively confirming a robust linear dose–response relationship. Throughout the exposure range, DBP levels exhibited a potential progressive, incremental trend with higher frequencies of takeaway consumption, and no evident threshold or saturation effect was observed. However, a cautious interpretation is warranted, as the 95% confidence intervals became relatively wide at higher exposure levels (≥6 times/week), suggesting increased statistical uncertainty in estimating the effect at these extreme frequencies. This exploratory visualization suggests that any potential impact of TFC on diastolic blood pressure among young adults might operate via a continuous trend rather than a threshold-triggered mechanism.

### 4.5. Mediation Analysis

**Figure 4:**
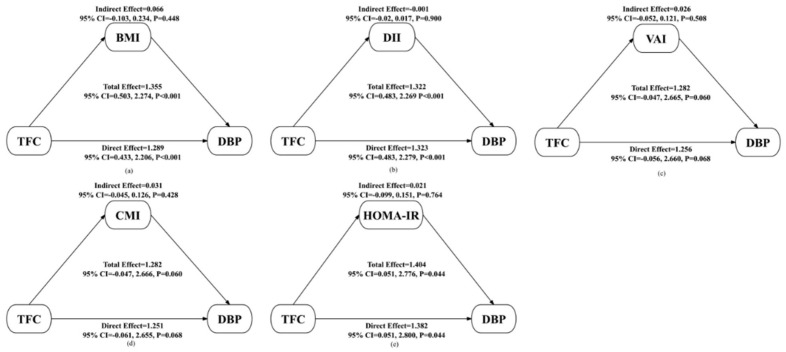
Analysis of the mediating roles of different markers in the relationship between takeaway fast-food consumption and diastolic blood pressure. Note: Exploratory mediation analyses of the association between takeaway fast-food consumption frequency and diastolic blood pressure. Path diagrams illustrate the estimated mediation effects of five potential metabolic and inflammatory indicators: BMI (**a**), DII (**b**), VAI (**c**), CMI (**d**), and HOMA-IR (**e**). Effect estimates for the Total Effect, Direct Effect, and Indirect Effect represent the absolute change in diastolic blood pressure (expressed in mmHg). Abbreviations: TFC, takeaway fast-food consumption; DBP, diastolic blood pressure; BMI, body mass index; DII, dietary inflammatory index; VAI, visceral adiposity index; CMI, cardiometabolic index; HOMA-IR, homeostasis model assessment of insulin resistance; CI, confidence interval. *p* < 0.05 was considered to indicate statistical significance.

Given the statistically association observed between high TFC frequency and elevated DBP specifically among females, we performed exploratory mediation analyses to investigate whether this relationship was statistically mediated by systemic inflammation or metabolic remodeling (Figure 4). We evaluated five established subclinical indices: BMI, DII, VAI, CMI, and HOMA-IR.

The ACMEs for all five indices were not statistically significant (all *p* > 0.05), indicating that neither generalized adiposity, visceral fat accumulation, systemic inflammation, nor insulin resistance significantly mediated the statistical association between TFC and DBP elevation in this young female cohort.

## 5. Discussion

In this nationally representative study of US adults, the main categorical association between the frequency of TFC and DBP did not reach statistical significance in the fully adjusted models. However, restricted cubic spline analysis revealed a potential continuous trend without an evident threshold effect. Furthermore, exploratory subgroup analyses identified a potentially pronounced, significant positive association specifically among females, although the formal interaction test was not statistically significant. Crucially, our exploratory mediation analyses demonstrated that this relationship was not significantly mediated by established metabolic or inflammatory indices, including BMI, visceral adiposity, and insulin resistance. Taken together, these findings suggest that the association between TFC and DBP elevation may exist independent of overt structural obesity, leaving a significant residual association that warrants further investigation.

Our observations help bridge the existing knowledge gap concerning the cardiovascular implications of contemporary eating behaviors. While numerous epidemiological studies have linked takeaway fast-food consumption to adiposity-related outcomes, such as increased BMI and waist circumference [8,29,30], the specific impact of TFC on blood pressure profiles remains under-investigated. Previous research has largely focused on the association between ultra-processed food intake—a major component of takeaway meals—and hypertension risk [9,31]. However, these studies often prioritized systolic blood pressure or aggregated hypertension endpoints, frequently failing to differentiate the distinct hemodynamic phenotype of diastolic blood pressure, which is of particular prognostic value in younger populations [3,4]. Furthermore, prior investigations have generally operated under the assumption that diet-induced hypertension is a secondary consequence of weight gain [10]. In contrast, our study utilizing restricted cubic spline analysis provides novel evidence of a linear dose–response relationship between TFC and DBP, with no apparent safety threshold. More critically, by employing an exploratory mediation framework, we observed that the potential association of TFC with DBP is not significantly mediated by established indices including BMI, visceral adiposity, and insulin resistance [32,33]. This distinction highlights a crucial, previously overlooked potential pathway driven by specific dietary constituents rather than metabolic accumulation. However, we strictly emphasize that this remaining statistical association should not be interpreted as a confirmed direct pathogenic mechanism.

Because our analyses revealed an association unmediated by adiposity, we hypothesize that the specific nutritional composition of takeaway fast-food might play a role, though these variables were not directly measured in our dataset. Quantitative analyses have repeatedly shown that takeaway meals—particularly fast foods and processed dishes—are exceptionally high in sodium [6,34]. Biologically, high sodium intake promotes water retention and extracellular volume expansion, which may influence systemic vascular resistance [12]. Furthermore, unmeasured dietary factors—such as direct endothelial effects of excess sodium [13], a low potassium-to-sodium ratio [9], inorganic phosphate additives [35], and industrially produced trans-fatty acids [36]—have been proposed in the literature as potential vascular stressors. Importantly, because sodium exposure and these specific dietary constituents were not directly assessed in our analytical models, these mechanistic explanations must be treated strictly as biologically plausible hypotheses requiring further empirical validation rather than mechanisms demonstrated by the present data.

The absence of significant mediation by traditional obesity metrics (BMI) and novel visceral adiposity indicators (VAI, CMI) in our study may initially seem counterintuitive, given the established link between excess adiposity and hypertension [10,11]. However, this finding remains conceptually plausible when considering the specific demographic characteristics of our study population. We focused exclusively on adults aged under 50 years, a group that likely possesses a higher degree of metabolic resilience compared to older cohorts. It is hypothesized that in this life stage, the vascular system may exhibit heightened sensitivity to dietary stressors commonly found in takeaway meals even in the absence of accumulation of visceral fat accumulation or the development of overt structural obesity [37]. Addressing the objective of early dietary intervention, these findings highlight the potential value of proactive dietary screening. Because blood pressure elevations may exist independent of overt metabolic dysfunction, intervening on dietary habits like TFC during this “pre-obesity” window could serve as an additional consideration in mitigating early-onset cardiovascular risks before structural metabolic derangements become clinically established [12,38].

Current hypertension prevention strategies and patient education materials predominantly emphasize weight management as the cornerstone of blood pressure control [33]. However, our findings offer preliminary insights that could complement these approaches. Specifically, our data suggest that assessing the frequency of meals prepared away from home might serve as a useful adjunct to routine dietary screening, even for young adults with a normal BMI. While clinicians traditionally focus on overt adiposity, screening for high-frequency takeaway fast-food consumption could help identify “hidden” cardiovascular risks before significant weight gain occurs [39]. From a public health perspective, rather than mandating immediate policy shifts, our findings broadly align with ongoing initiatives aimed at improving nutritional transparency and food formulation. Nevertheless, these clinical and public health perspectives remain hypothesis-generating and strictly require confirmation in prospective longitudinal or intervention studies.

The robustness of our analysis stems from several distinct merits inherent in the study design. First, it utilized a large, nationally representative sample from NHANES, ensuring high statistical power and generalizability to the broader US young adult population [14]. Second, unlike previous studies that focused solely on gross associations, we employed exploratory mediation analyses with multiple metabolic indicators to dissect the specific pathways linking takeaway fast-food to blood pressure. Third, by focusing on diastolic blood pressure in adults under 50 years, we targeted a critical yet often overlooked window for early cardiovascular prevention.

Nonetheless, several inherent limitations must be explicitly acknowledged. First and foremost, the observational, cross-sectional nature of this dataset restricts our ability to establish temporal precedence, thus limiting definitive conclusions regarding a causal link. Because we cannot confirm the temporal ordering between exposure, mediators, and the outcome, the mediation findings should never be interpreted as evidence of a confirmed direct vascular mechanism. Furthermore, it is crucial to interpret these exploratory mediation analyses with particular caution, given that the overall categorical association between TFC and DBP did not reach statistical significance in the fully adjusted model. The absence of significant mediation by the examined indices should not be misconstrued as definitive evidence supporting alternative direct biological pathways. Instead, the residual associations highlight the possibility of unmeasured confounding, such as high sodium intake. Additionally, reverse causality must be explicitly considered; individuals who had already developed elevated blood pressure might have subsequently modified their dietary habits, which could profoundly influence the observed associations. Second, dietary data were derived from self-reported 24-h recalls and the use of a single questionnaire item to assess takeaway fast-food consumption frequency. These self-reported metrics are subject to recall bias and may not accurately reflect long-term or habitual dietary patterns. Furthermore, relying exclusively on a single questionnaire item inherently restricts our assessment to mere consumption frequency, lacking the necessary granularity to capture critical nuances such as portion sizes, specific meal components, and the precise nutritional quality of the fast food consumed. Nevertheless, the overall dietary assessment in NHANES utilizes the Automated Multiple-Pass Method (AMPM) and has been validated for population-level assessment [40]. Third, blood pressure was measured during a single visit, which may not fully account for day-to-day variability. Fourth, regarding the restricted cubic spline analysis, we note that the 95% confidence intervals became relatively wide at higher exposure levels, which strictly limits the precision of estimates in this extreme range. Finally, even with meticulous adjustment for diverse parameters, the possibility of unmeasured confounding influence from non-included lifestyle variables remains a consideration.

## 6. Conclusions

In conclusion, while the overall main categorical association did not reach statistical significance, our exploratory analyses suggest a potential continuous trend between takeaway fast-food consumption frequency and elevated diastolic blood pressure among US adults aged under 50 years. Furthermore, this association was observed to be potentially pronounced within the female subpopulation. Notably, this statistical association is not significantly mediated by generalized obesity (BMI), visceral adiposity (VAI, CMI), or diet-related systemic inflammation (DII). Instead, these findings suggest that the association between takeaway fast-food and blood pressure elevation may exist independent of overt structural obesity. Consequently, assessing takeaway fast-food consumption frequency could serve as a useful preliminary consideration for early cardiovascular prevention, regardless of an individual’s body weight or metabolic status. However, given the cross-sectional design of our study, these findings and their clinical and public health implications are hypothesis-generating and strictly require confirmation in prospective longitudinal or intervention studies.

## Figures and Tables

**Table 1:** Baseline characteristics of the study population according to quartiles of takeaway fast-food consumption (NHANES 2009–2018, N = 13,062).

		TFC Frequency				
Characteristics	Overall	Q1 (0–1)	Q2 (2–3)	Q3 (4–5)	Q4 (≥6)	*p*-Value
N (%)	13,062 (100%)	5247 (40.17%)	4018 (30.76%)	2269 (17.37%)	1528 (11.70%)	
Age, years	34.88 (9.04)	35.44 (8.95)	34.81 (9.02)	34.45 (9.12)	34.01 (9.09)	0.001
Sex, *n* (%)						<0.001
Male	6302 (49.8)	2460 (48.8)	1786 (45.3)	1160 (52.9)	896 (59.4)	
Female	6760 (50.2)	2787 (51.2)	2232 (54.7)	1109 (47.1)	632 (40.6)	
Race, *n* (%)						<0.001
Mexican American	2121 (11.5)	947 (13.1)	657 (11.5)	311 (9.6)	206 (9.4)	
Other Hispanic	1348 (7.5)	593 (8.4)	422 (7.9)	202 (6.4)	131 (5.8)	
Non-Hispanic White	4737 (59.1)	1788 (55.9)	1501 (59.6)	861 (62.0)	587 (63.2)	
Non-Hispanic Black	2727 (12.2)	960 (11.3)	853 (12.3)	558 (13.6)	356 (12.2)	
Other Race—Including Multi-Racial	2129 (9.8)	959 (11.4)	585 (8.7)	337 (8.4)	248 (9.4)	
Educational attainment, *n* (%)						<0.001
Less than 9th grade	843 (4.3)	493 (6.2)	217 (3.8)	77 (2.4)	56 (2.5)	
9–11th grade (Includes 12th grade with no diploma)	1722 (10.1)	809 (12.1)	507 (9.3)	241 (8.8)	165 (8.2)	
High school graduate/GED or equivalent	2814 (21.4)	1136 (22.1)	900 (22.0)	475 (20.5)	303 (18.9)	
Some college or AA degree	4290 (33.2)	1586 (32.4)	1384 (34.9)	798 (33.5)	522 (31.5)	
College graduate or above	3393 (31.1)	1223 (27.3)	1010 (30.1)	678 (34.9)	482 (39.0)	
Poverty income ratio	2.83 (1.66)	2.60 (1.68)	2.81 (1.64)	3.08 (1.63)	3.15 (1.64)	<0.001
Weight, kg	83.38 (22.47)	82.75 (22.33)	82.90 (22.63)	84.89 (22.91)	84.18 (21.78)	0.019
Height, cm	169.59 (9.84)	168.98 (9.91)	169.04 (9.80)	170.37 (9.59)	171.60 (9.77)	<0.001
Waist circumference, cm	97.18 (16.94)	97.14 (16.74)	96.95 (17.03)	97.87 (17.27)	96.83 (16.77)	0.395
Pulse, beats/min	73.73 (11.70)	73.95 (11.69)	73.85 (12.10)	73.44 (11.49)	73.19 (11.01)	0.255
Systolic blood pressure, mmHg	116.71 (13.14)	116.79 (13.47)	116.42 (13.17)	116.79 (13.07)	117.03 (12.18)	0.632
Diastolic blood pressure, mmHg	71.03 (11.12)	71.12 (11.35)	70.67 (10.87)	71.15 (11.09)	71.49 (11.08)	0.192
Hemoglobin A1c, %	5.42 (0.79)	5.45 (0.84)	5.42 (0.76)	5.39 (0.72)	5.41 (0.80)	0.054
Total cholesterol, mmol/L	4.88 (1.03)	4.93 (1.08)	4.87 (1.01)	4.85 (1.01)	4.84 (0.98)	0.098
High-density lipoprotein cholesterol, mmol/L	1.35 (0.40)	1.35 (0.40)	1.36 (0.40)	1.36 (0.40)	1.35 (0.38)	0.576
Creatinine, umol/L	74.44 (24.15)	74.02 (28.66)	73.10 (19.00)	75.54 (25.52)	77.34 (17.47)	<0.001
Body mass index	28.90 (7.10)	28.89 (7.05)	28.92 (7.21)	29.16 (7.22)	28.52 (6.79)	0.211
Smoking status						0.989
Yes	4984 (39.8)	2013 (39.9)	1512 (39.6)	872 (40.1)	587 (39.6)	
No	8078 (60.2)	3234 (60.1)	2506 (60.4)	1397 (59.9)	941 (60.4)	
Vigorous work activity						0.505
Yes	3111 (25.4)	1203 (25.5)	987 (25.7)	565 (26.4)	356 (23.0)	
No	9950 (74.6)	4044 (74.5)	3031 (74.3)	1703 (73.6)	1172 (77.0)	
Moderate work activity						0.084
Yes	5324 (43.7)	2073 (43.0)	1660 (44.4)	993 (46.5)	598 (40.3)	
No	7737 (56.3)	3173 (57.0)	2358 (55.6)	1276 (53.5)	930 (59.7)	
Hypertension						0.457
Yes	9383 (73.1)	3755 (73.0)	2851 (72.2)	1662 (73.5)	1115 (74.8)	
No	3679 (26.9)	1492 (27.0)	1167 (27.8)	607 (26.5)	413 (25.2)	
Fasting subsample indicators						
N (%)	5574 (100%)	2225 (39.92%)	1718 (30.82%)	995 (17.85%)	636 (11.41%)	
Fasting glucose, mmol/L	5.63 (1.46)	5.64 (1.52)	5.60 (1.43)	5.60 (1.36)	5.71 (1.52)	0.305
Serum insulin, pmol/L	76.19 (89.20)	74.92 (82.78)	75.49 (75.14)	75.96 (65.63)	82.12 (151.00)	0.738
Triglycerides, mmol/L	116.61 (110.64)	112.76 (82.69)	117.27 (143.88)	121.81 (102.69)	118.26 (100.82)	0.145
Low-density lipoprotein cholesterol, mmol/L	2.89 (0.86)	2.92 (0.88)	2.89 (0.86)	2.86 (0.87)	2.87 (0.85)	0.586
Homeostatic Model Assessment for Insulin Resistance	2.95 (5.01)	2.96 (5.14)	2.91 (4.11)	2.84 (2.81)	3.23 (8.31)	0.618
Visceral adiposity index	1.86 (2.55)	1.79 (1.91)	1.92 (3.37)	1.92 (2.16)	1.85 (2.44)	0.204
Cardiometabolic index	0.70 (1.03)	0.67 (0.78)	0.71 (1.31)	0.74 (0.89)	0.72 (1.09)	0.157

Note: Continuous variables are presented as weighted means (standard deviation, SD), and categorical variables are expressed as unweighted frequencies (weighted percentages). To ensure national representativeness and account for the complex, multistage probability sampling design of NHANES across the combined survey cycles (2009–2018), the full-sample Mobile Examination Center (MEC) weights were applied to demographic, socioeconomic, anthropometric, and routine clinical variables. Conversely, metabolism-related indicators exclusively measured in the randomly assigned morning fasting subsample (including fasting glucose, serum insulin, triglycerides, low-density lipoprotein cholesterol, homeostatic model assessment for insulin resistance, visceral adiposity index, and cardiometabolic index) were strictly estimated utilizing the designated fasting subsample weights. Differences across TFC quartiles were assessed using the survey-weighted Wald test for continuous variables and the Rao–Scott Chi-square test for categorical variables.

**Table 2:** Association between takeaway fast-food consumption frequency and the prevalence of hypertension (NHANES 2009–2018).

	Model 1		Model 2		Model 3	
	OR (95% CI)	*p*	OR (95% CI)	*p*	OR (95% CI)	*p*
Q1 (0–1)	1.00 (Ref)		1.00 (Ref)		1.00 (Ref)	
Q2 (2–3)	1.04 (0.92, 1.18)	0.538	1.07 (0.94, 1.21)	0.328	1.07 (0.93, 1.23)	0.342
Q3 (4–5)	0.97 (0.85, 1.10)	0.64	1.00 (0.88, 1.15)	0.967	1.04 (0.90, 1.20)	0.633
Q4 (≥6)	0.91 (0.76, 1.09)	0.294	0.96 (0.80, 1.16)	0.684	1.03 (0.84, 1.27)	0.744
*p* for trend	0.27		0.72		0.666	

Note: Values are presented as odds ratios (95% confidence intervals) derived from survey-weighted logistic regression models. Model 1: Unadjusted. Model 2: Adjusted for age, sex, and race. Model 3: Further adjusted for education, poverty income ratio, smoking status, physical activity, and body mass index. Abbreviations: OR, odds ratio; CI, confidence interval; Ref, reference category.

**Table 3:** Association between takeaway fast-food consumption frequency and diastolic blood pressure levels (NHANES 2009–2018).

	Model 1		Model 2		Model 3	
	β (95% CI)	*p*	β (95% CI)	*p*	β (95% CI)	*p*
Q1 (0–1)	0.00 (Ref)	-	0.00 (Ref)	-	0.00 (Ref)	-
Q2 (2–3)	−0.46 (−1.08, 0.17)	0.147	−0.11 (−0.69, 0.47)	0.701	−0.09 (−0.66, 0.49)	0.766
Q3 (4–5)	0.03 (−0.62, 0.67)	0.933	0.18 (−0.42, 0.78)	0.546	−0.02 (−0.66, 0.62)	0.954
Q4 (≥6)	0.36 (−0.45, 1.18)	0.378	0.45 (−0.37, 1.28)	0.276	0.53 (−0.29, 1.36)	0.201
*p* for trend	0.422		0.266		0.327	

Note: Values are presented as β coefficients (95% confidence intervals) derived from survey-weighted linear regression models. Model 1: Unadjusted. Model 2: Adjusted for age, sex, and race. Model 3: Further adjusted for education, poverty income ratio, smoking status, physical activity, and body mass index. Abbreviations: CI, confidence interval; Ref, reference category.

**Table 4:** Association between takeaway fast-food consumption frequency and systolic blood pressure levels (NHANES 2009–2018).

	Model 1		Model 2		Model 3	
	β (95% CI)	*p*	β (95% CI)	*p*	β (95% CI)	*p*
Q1 (0–1)	0.00 (Ref)	-	0.00 (Ref)	-	0.00 (Ref)	-
Q2 (2–3)	−0.38 (−1.15, 0.40)	0.334	−0.01 (−0.74, 0.72)	0.973	0.15 (−0.58, 0.88)	0.686
Q3 (4–5)	−0.01 (−0.79, 0.77)	0.986	−0.13 (−0.87, 0.61)	0.729	−0.16 (−0.92, 0.60)	0.68
Q4 (≥6)	0.23 (−0.63, 1.10)	0.592	−0.14 (−0.94, 0.66)	0.731	0.16 (−0.64, 0.96)	0.687
*p* for trend	0.649		0.659		0.935	

Note: Values are presented as β coefficients (95% confidence intervals) derived from survey-weighted linear regression models. Model 1: Unadjusted. Model 2: Adjusted for age, sex, and race. Model 3: Further adjusted for education, poverty income ratio, smoking status, physical activity, and body mass index. Abbreviations: CI, confidence interval; Ref, reference category.

## Data Availability

The datasets supporting the conclusions of this article are available in the National Health and Nutrition Examination Survey (NHANES) repository, unique persistent identifier and hyperlink to datasets in https://wwwn.cdc.gov/nchs/nhanes/Default.aspx (accessed on 15 April 2026).

## Abbreviations

TFC: takeaway fast-food consumption; DBP: diastolic blood pressure; NHANES: National Health and Nutrition Examination Survey; RCS: Restricted cubic splines; BMI: body mass index; VAI: visceral adiposity index; CMI: cardiometabolic index; DII: dietary inflammatory index; HOMA-IR: Homeostasis Model Assessment for Insulin Resistance; CVD: cardiovascular disease; IDH: isolated diastolic hypertension; NCHS: National Center for Health Statistics; MECs: Mobile Examination Centers; WC: waist circumference; TG: triglycerides; HDL-C: high-density lipoprotein cholesterol; WHtR: waist-to-height ratio; PIR: income-to-poverty ratio; FG: fasting glucose; TC: total cholesterol; LDL-C: low-density lipoprotein cholesterol; CIs: confidence intervals; ORs: odds ratios; TE: total effect; ADE: average direct effect; ACME: average causal mediation effect; SBP: Systolic Blood Pressure; AMPM: Automated Multiple-Pass Method.
